# The Western Hahnemannian Club

**Published:** 1893-05

**Authors:** Horace P. Holmes

**Affiliations:** Omaha, Neb.


### THE WESTERN HAHNEMANNIAN CLUB.

HORACE P. HOLMES, M. D., OMAHA, NEB.

At the office of Dr. H. P. Holmes, in Omaha, there gathered Saturday evening, February 11th, a number of physicians for the purpose of forming a medical society, the principles of which should be strictly Hahnemannian. It is the first institution of the kind west of Chicago, and the event certainly marks an era in the work and spread of the better kind of Homœopathy. Ten physicians joined as charter members and several more have promised, so the new society enters upon its career with some sixteen members.

A peculiar feature of the meeting was the perfect unanimity of feeling, as not a sentence of the preamble, declaration, Constitution, nor By-Laws met with an opposing voice. The only feeling manifested was that the new society should be as perfectly formed and governed as was necessary for an institution of the kind.

The following preamble and declaration was adopted:

"In consequence of the fact that Homœopathy, as taught by Hahnemann through his life-work and his *Organon*, is suffering at the hands of those who would adulterate it with the uncertainties of allopathy, electicism, and empiricism, we, the undersigned physicians, believing that the truest and best system of therapeutics is that covered by the law 'Similia Similibus Curantur' and the *Organon* to be the best guide in practice, do hereby organize ourselves into the *Western Hahnemannian Club* for the purpose of promoting the welfare of true Homœopathy and for our mutual improvement."

"Declaration: We believe the principles set forth in Hahnemann's *Organon of the Healing Art* to be the only true guide in therapeutics, the law of similars the only basis for prescribing, the single indicated remedy to be ever the best, and that as practitioners of Homœopathy we consider the mixing and alternating of remedies to be non-homœopathic, and believe that the nearer we follow the law of similia the better will be the results."

The Constitution provides for the application and admittance of members in the following manner: The applicant shall indorse the declaration at the time his application is presented. This application shall lay over for one month, and at that time is voted upon by ballot only. Two negative ballots shall lay it over for one month more, and at this second trial two negative ballots shall lay it over for six months. No application shall be presented to the society nor be acted upon with the applicant present.

It is the aim and intention of the club to be largely of a social nature and to run with as little machinery as possible. But one permanent officer is elected, that one being the Secretary, who shall also act as Treasurer. The presiding officer is temporarily appointed at each meeting. The club will start with Omaha and Council Bluffs as a nucleus, and it is hoped it will rapidly grow to take in a large portion of the Missouri Valley. At present its meetings will be held semi-monthly, the first and third Saturday evenings in each month. Quarterly or semi-annually extra sessions will be held, at which time outside members will have a better opportunity to be in attendance.

